# Genetic Rat Models of Parkinson's Disease

**DOI:** 10.1155/2012/128356

**Published:** 2012-04-05

**Authors:** Ryan M. Welchko, Xavier T. Lévêque, Gary L. Dunbar

**Affiliations:** ^1^Field Neurosciences Institute, Laboratory for Restorative Neurology, Department of Psychology, Central Michigan University, Mt. Pleasant, MI 48859, USA; ^2^Field Neurosciences Institute, Laboratory for Restorative Neurology, Program in Neuroscience, Central Michigan University, Mt. Pleasant, MI 48859, USA; ^3^Field Neurosciences Institute, Laboratory for Restorative Neurology, Department of Psychology and Program in Neuroscience, College of Medicine, Central Michigan University, Mt. Pleasant, MI 48859, USA; ^4^Field Neurosciences Institute, St. Mary's of Michigan, Saginaw MI 48604, USA

## Abstract

Parkinson's disease (PD) is a neurodegenerative disease characterized by a specific loss of dopaminergic neurons. Although the vast majority of PD cases are idiopathic in nature, there is a subset that contains genetic links. Of the genes that have been linked to PD, *α*-synuclein and leucine-rich repeat kinase 2 have been used to develop transgenic rat models of the disease. In this paper we focused on the various transgenic rat models of PD in terms of their ability to mimic key symptoms of PD in a progressive manner. In general, we found that most of these models provided useful tools for the early stages of PD, but the development of new transgenic rats that present significant neuropathologic and motoric deficits in a progressive manner that more accurately mimics PD is needed.

## 1. Introduction

Parkinson's disease (PD) is the second most common neurodegenerative disorder. It is hypothesized that PD is caused by a combination of genetic and environmental factors. A specific loss of dopaminergic (DA) neurons in the substantia nigra (SN) in PD results in the significant loss of DA innervation of the neostriatum via the nigrostriatal pathway. The progressive loss of this DA innervation, in turn, is responsible for the characteristic motor symptoms of PD, which include akinesia, resting tremors, and postural instability.

A hallmark histopathological marker for PD is the presence of cytoplasmic inclusions, known as Lewy bodies (LBs), which are found throughout the mesencephalon of the PD brain, including the substantia nigra [1]. The major component of LBs is the presynaptic protein called *α*-synuclein (*α*-syn) [2]. To date, it is not known whether the mechanisms underlying the formation of LBs are the cause or a consequence of the disease. Some familial forms of PD have been linked to mutations in genes that code for *α*-syn, as well as genes that code for leucine-rich repeat kinase 2 (LRRK2), DJ-1, and Parkin [3, 4]. These mutations have formed the basis of developing transgenic rodent models of PD.

Presently, the only treatments for PD are palliative in nature and usually utilize dopamine agonists, such as levodopa (L-DOPA). Surgical techniques, such as pallidotomies and deep-brain stimulation, have also proved to be successful in reducing some of the major motor symptoms of PD. To date, the most promising avenue of research aimed at restoring lost function in PD appears to be cell therapies, and much of the recent research efforts in this domain have focused on use of stem cells.

However, significant advances in developing effective therapies for PD have been limited by the lack of a valid animal model that closely mimic the progressive neuropathology and behavioral deficits of PD. Most research on PD has utilized rodents that are given specific neurotoxins, including 6-hydroxydopamine (6-OHDA), 1-methyl-4-phenyl-1,2,3,6-tetrahydropyridine (MPTP), paraquat, and rotenone, that are administrated into the substantia nigra pars compacta (SNpc) or nigrostriatal pathway. Although these models have yielded valuable insights into potentially useful therapies for PD, they do not accurately mimic the full constellation of neuropathological and behavioral deficits associated with PD. Recently, transgenic animal models of PD have been developed by inserting the mutated human gene in rodents. Although most of these transgenic models have been developed in mice, more recent efforts have focused on developing transgenic rat models of PD.

The purpose of this paper is to provide an update on the newly developed genetic rat models of PD and to provide some assessment of their usefulness in therapeutic research. We will attempt to compare each of the newly developed models in terms of their ability to mimic both the neuropathological and behavioral features of the disease (see Table 1). We will also describe some of the early therapeutic research using these models. Although none of these models provide the optimal tool for testing potential treatments for PD, we think that some of these models have the potential to provide an improvement over the more commonly used neurotoxic rat models.

## 2. *α*-Synuclein Rat Models

The most thoroughly studied transgenic rat model of PD is the *α*-synuclein rat model. As indicated previously, some familial forms of PD have been linked to *α*-syn, a small neuronal 140 amino acid protein (14.4 kDa) [5], that is located in presynaptic terminals. In humans, the gene for *α*-syn is located on chromosome 4q21-q23 [6] and this protein is highly expressed in the brain. Missense autosomal dominant mutations at A53T [7], E46K [8], and A30P [9] of the gene-coding region of *α*-syn have been linked to LB aggregates and Lewy neurites in patients with rare, early onset PD. The presynaptic presence of *α*-syn suggests a role in synaptic vesicle trafficking. It has been shown that *α*-syn is a dose-dependent inhibitor of tyrosine hydroxylase (TH) [10]. On the other hand, *α*-syn could be a link in the dopamine transporter and, in this way, increase dopamine reuptake [11]. As such, it appears that an optimal level of *α*-syn may be critical for proper functioning of DA neurons.

Transgenic *α*-syn models have been created in adult rats utilizing adenoassociated virus (rAAV) or HIV-1-derived lenti-virus to transduce mutated human *α*-syn genes into the nigrostriatal pathway. Also, HIV TAT-mediated protein transduction has been used to transduce mutated human *α*-syn protein. Although there has been some success with achieving expression of *α*-syn in DA neurons within the SN, the amount of DA neuronal degeneration varied greatly between studies assessed by TH immunohistochemistry (IHC). Some of this variation could be due to differing forms of the *α*-syn gene used, the method of delivery utilized, or the gene promoter used. For example, Klein et al. [13] found a 53% reduction of TH-positive DA neurons when they injected rAAV expressing the mutant human *α*-syn A30P into the SN, while Kirik et al. [12] reported a 30–80% reduction when they injected rAAV expressing the mutant human *α*-syn A53T in the SN. Klein et al. [13] and Kirik et al. utilized a hybrid cytomegalovirus (CMV)/chicken *β*-actin (CBA) promoter to drive expression of the differing mutant *α*-syn genes. This specific promoter is the CBA promoter with enhancer elements from the CMV promoter added. In addition, Kirik et al. [12] found a 50% reduction in TH positive DA striatal innervation at eight weeks after injection. In term of behavior, Kirik et al. [12] reported that 25% of the *α*-syn-injected rats were impaired during apomorphine-induced rotation assessments and on a paw-reaching (i.e., cylinder) task, an impairment that is compatible to a loss of more than 60% of DA SN neurons. However, Klein et al. [13] did not find a significant behavioral impairment in amphetamine-induced rotations. Both Kirik and Klein groups of investigators reported progressive pattern of neurodegeneration that included *α*-syn aggregates that resembled LBs in PD patients. As such, these transgenic rat models of PD more closely mimicked the progressive loss of dopaminergic neurons in PD than do the neurotoxic rat models of this disease. 

Yamada et. al [15] created an *α*-syn model using rAAV, but utilized a cytomegalovirus (CMV) promoter which differed from the CMV/CBA promoter used by the Kirik and Klein groups [12, 13]. Yamada et al. reported a 50% decrease in the number of DA neurons in the injected SN, but this decrease was insufficient to produce significant differences between the injected rats and controls when these animals were assessed on measures of apomorphine-induced rotation.

Recchia et al. [18] created an *α*-syn model of PD by administering intranigral injections of the A30P mutated form of *α*-syn, fused to a TAT fusion protein. Their study compared unilateral injections of TAT-*α*-syn A30P protein, *α*-syn A30P protein, 6-OHDA, and sham controls into the SN at various time points. Neurodegeneration and genetic expression were assessed via IHC, western blot, HPLC analysis, and behavioral assessment. They found that the *α*-syn A30P protein, without the TAT fusion protein, did not integrate within neurons of the SN, whereas 80% of TH-positive cells were found to express *α*-syn A30P following TAT-*α*-syn A30P injection. No formations of LBs or Lewy neurites were found in these animals. The TAT-*α*-syn A30P distribution, assessed by western blot, was found within the SN, striatum, and contralateral cortex at 24 hours after injection. At 15 and 30 days post-injection, the TAT-*α*-syn A30P produced a 26.3 ± 5.0% loss in TH positive neurons, compared to the 81.2 ± 2.1% loss in TH positive neurons following the 6-OHDA injections. At 7 days post-injection, the TAT-*α*-syn A30P animals showed a 20.8 ± 3.6% reduction in DA and 16.6 ± 2.5% reduction in 3,4-dihydroxyphenylacetic acid (DOPAC) within the ipsilateral, compared to the contralateral, striatum. In comparison, the animals receiving 6-OHDA injections showed a 60.6 ± 3.6% reduction in DA and a 59.7% ± 4.5 reduction in DOPAC within the ipsilateral, compared to the contralateral, striatum. Administration of apomorphine did not produce a significant number of ipsilateral rotations in animals receiving TAT-*α*-syn A30P injections. However, rats receiving TAT-*α*-syn A30P injections showed increasing motor impairment at day 30 and day 90 when motor function was accessed via rotorod and footprint analysis. 

 More recently, Sanchez-Guajardo et al. [22] studied the temporal profile of microglia activation in an *α*-syn rat model of PD. They injected rAAV2/5 expressing human wild type (hWT) *α*-syn directly into the right SN. Rats were divided into two groups based upon the concentration of rAAV2/5 hWT *α*-syn. The *α*-syn neurodegeneration group received a lower concentration of rAAV2/5 hWT *α*-syn, while the *α*-syn cell-death group received a greater concentration. The *α*-syn neurodegeneration rats did not exhibit a significant reduction in TH-positive neurons within the SN. However, a decrease in TH-positive fiber density within the striatum and pathological formations were observed in these animals. The *α*-syn cell-death group exhibited a significant reduction in TH-positive neurons within the SN and a reduction of TH-positive fiber density within the striatum. An increase in microglia was seen in both groups at four weeks after injection. The activated microglia was significantly higher in the neurodegeneration group at 4 weeks, and the levels of microglia returned to control levels at 8–15 weeks. Within the cell-death group, microglia levels peaked at 8 weeks and returned to control levels at 15 weeks. 

 Lelan et al. [19] created a transgenic *α* syn rat utilizing microinjection to transfer the construct pUTHTV expressing the A30P and A53T double-mutated human *α* syn under the control of the rat TH promoter. These investigators observed the expression of mutated human *α*-syn in olfactory bulbs and within SN. They also observed an olfactory impairment in mutated human *α* syn transgenic rats, when compared to wild type rats. No impairment in motor behavior was observed. 

 Gorbatyuk et al. [16] created three *α*-syn models with the hWT *α*-syn or mutated *α*-syn with S129A, or S129D mutations. Unilateral injections of rAVV2/5 vectors expressing the transgenes were done in the SN. Histological examinations were carried out at 4, 8, or 26 weeks after injection. At 4 weeks, a 70% loss in TH-positive neurons was observed in the rats receiving S129A, whereas all other groups did not show a significant TH-positive neuronal loss, when compared to controls. At 8 weeks, rats receiving an injection of WT *α*-syn rats exhibited a 40% loss of TH-positive neurons and S129A *α*-syn rats exhibited a 66% loss of TH neurons. However, rats receiving hWT *α*-syn exhibited a 60% loss of TH positive neurons at 27 weeks, which was similar to the S129A *α*-syn rats at that time point. Rats receiving the S129D injections did not exhibit significant neurodegeneration at any of the time points. Striatal DA levels were assessed by HPLC. The HPLC analysis showed that there was a depletion of DA levels, consistent with SN cell loss. 

 Azeredo da Silveira et al. [17] created an *α*-syn model to observe a prevention of the phosphorylation of human mutated *α*-syn which is involved in numerous neurodegenerative diseases. The study utilized the rAAV2/6 vector with the CMV promoter. The site-directed mutations of human A30P *α*-syn and hWT *α*-syn were at the serine residue at position 129. The serine residue was converted to alanine (S129A) to abolish phosphorylation or converted to aspartate (S129D) to reproduce effects of phosphorylation. The two site-directed mutations of the mutated human A30P *α*-syn and hWT *α*-syn were compared to hWT *α*-syn and mutated human A30P *α*-syn. Rats received two injections within the SN. They observed a dose-dependent loss from 11 to 22% in TH-positive neurons when rats received injections of hWT *α*-syn. Injection of the S129A-Mutated A30P *α*-syn and hWT *α*-syn resulted in a dose-dependent loss of over 70%. Whereas the S129D-mutated A30P *α*-syn and human WT *α*-syn resulted in less neurodegeneration in the SN then WT *α*-syn, rats receiving injections of hWT *α*-syn with the mutated human A30P *α*-syn tended to display less neurodegeneration than the WT *α*-syn rats. 

Finally, a series of *α*-syn models of PD were produced by Lo Bianco et al. [14], using an HIV-1-derived lenti-virus, expressing a range of *α*-syn genes, including wild-type human *α*-syn, mutated-human A30P *α*-syn, mutated-human A53T *α*-syn, and rat wild-type *α*-syn. The largest reduction of TH-positive neurons within the SN was observed in animals treated with the lenti-WT human *α*-syn, which exhibited a 35% reduction. Rats treated with A30P *α*-syn and A53T *α*-syn also exhibited a 33% and 24% reduction in TH-positive neurons, respectively. Some *α*-syn inclusions were found in the cytoplasm of neurites and cell bodies of surviving nigral neurons. 

Subsequent studies using genes that overexpressed glial derived neurotrophic factor (GDNF), a protein associated with neuroprotection of dopaminergic neurons, were unsuccessful in reducing the cell loss in several of these *α*-syn rat models of PD. For example, Lo Bianco et al. [23] found that injections of a lenti-GDNF just dorsal to the SN, given two weeks prior to bilateral injections of lenti-A30P-*α*-syn, failed to reduce the loss of DA neurons as assessed by TH immunohistochemistry. Similarly, Decressac et al. [24] injected lenti-GDNF into the striatum two weeks prior to an intranigral injection of rAAV2-A30P-*α*-syn and, in a second study, injected rAAV2-GDNF in the striatum and just dorsal to the SN at three weeks prior to an intranigral injection of rAAV2-A30P-*α*-syn and found that these GDNF injections failed to protect against *α*-syn-induced neurotoxicity in both studies. 

 Lo Bianco et al. also tested the potential efficacy of using lenti-viral delivery of parkin in an *α*-syn rat model of PD [25]. Parkin is a 465-amino-acid protein responsible for protein degradation, and mutated forms of parkin are found in about 50% of the cases of juvenile PD, an autosomal recessive form of this disease [26]. Lo Bianco et al. gave two groups of rats bilateral injections into the right SN of either lenti-A30P-*α*-syn/lenti-YFP, or lenti-A30P-*α*-syn/lenti-Parkin (with the Parkin gene being derived from a wild-type rat). Control animals received injections of either lenti-Parkin or lenti-YFP. A 31% reduction of TH-positive neurons in the SN was observed in animals receiving lenti-A30P-*α*-syn, whereas a 9% reduction in TH positive neurons was observed in the SN of animals receiving lenti-A30P-*α*-syn with lenti-Parkin. There was a 16% reduction in TH-positive neurites within the striatum of the lenti-A30P-*α*-syn rats. The animals receiving lenti-A30P-*α*-syn with lenti-Parkin exhibited a 4% reduction in TH-positive neurites within the striatum. In addition, animals receiving lenti-A30P-*α*-syn with lenti-Parkin, as evidenced by silver staining, did not show an *α*-syn-induced neurodegeneration, whereas the lenti-A30P-*α*-syn group did. Animals receiving lenti-A30P-*α*-syn with lenti-Parkin exhibited a 45% increase in hyperphosphorylated *α*-syn inclusion, and lenti-A30P- *α*-syn animals showed a 43% increase in hyperphosphorylated *α*-syn inclusions. Similarly, Yamada et al. [4] examined the effects of Parkin on the rAAV *α*-syn model of PD. They injected rAAV expressing *α*-syn and rAAV parkin at the same time directly into SN and observed a decrease of cell death of DA neurons within the SN and the number of apomorphine-induced rotations, compared to rat injected with rAAV *α*-syn alone. Clearly, the *α*-syn rat models of PD provide many of the features that resemble symptoms of PD. However, most of the models produce a relatively limited cell loss, which provides a useful model of early PD but lacks the extensive cell loss and behavioral deficits to accurately mimic the later stages of this disease. Early therapeutic trials in these rat models have suggested that overexpression of the gene for parkin, but not GDNF, may reduce TH-positive cell loss in this model of PD. 

## 3. 4-Leucine-Rich Repeat Kinase 2 (LRRK2)

A second type of transgenic rat model involves the use of LRRK2, which is a member of the ROCO protein family and the RAS-GTPase super family. The LRRK2 gene contains both a GTPase-like domain and a kinase-like domain. An important number of mutations in the gene have been identified and associated with PD. Of these mutations, G2019S has been found to be the most common [27, 28] and is a point mutation located within the kinase domain. Another common mutation is R1441C, in which a mutation occurs within the GTPase domain. 

 Zhou et al. [21] utilized LRRK2 transgenic rats possessing the G2019S mutation. Transgenic rats were created by crossing TRE-LRRK2 G2019S transgenic rats with CAG-tTA transgenic rats. These investigators examined the effects of temporal expression of the human LRRK2 mutation, using a promoter that is activated by the absence of doxycycline. They measured the total distance and stereotypical movements the rats made in an open-field maze during a 20-minute assessment period, followed by an addition 20-minute period, which was given 5 minutes after an injection of amphetamine or 20 minutes after an injection of nomifensine (an inhibitor of DA reuptake). They found that the temporal expression of the human LRRK2 G2019S increased the distance traveled as well as the amount of stereotypical behavior. When given nomifensine or amphetamine, the temporal expression of LRRK2 G2019S significantly reduced the amount of evoked responses, compared to controls. In addition to the open-field assessments, these investigators measured intrastriatal DA via microdialysis and found an increase in extracellular DA in the striatum of rats receiving temporal expression of LRRK2. However, there were no increase in the level of DA when amphetamine and nomifensine was administered to these rats. The researchers attributed these results to an impairment of the DA transporter (DAT) due to preservation of in TH-positive neurons within the SNpc, nor were there any changes observed in the number of axon terminals in the striatum. 

 Dusonchet et al. [20] produced a transgenic LRRK2 model in adult rats utilizing recombinant human serotype 5 adenovirus (RAd). These investigators compared the overexpression of human wild-type LRRK2 to the LRRK2 G2019S human mutation. They found no loss of TH-positive cells in the human wild-type LRRK2 animals. However, there was a progressive TH-positive cell loss, observed between 10 and 42 days (with a total loss of 21.4%), and a 10% decrease in striatal TH-positive fibers in G2019S LRRK2 animals. 

 As with the *α*-syn models, the LRRK2 transgenic rat models of PD appear to provide only a modest loss of TH-positive cells. However, there is evidence of motor deficits, as measured in the open-field maze, when the LRRK2 G2019S gene is activated in this transgenic rat model of PD. As such, this model may also provide a useful tool for evaluating potential treatments during the early stages of PD.

## 4. Conclusion

In this paper, we have provided an update on the various genetic rat models of PD. Although there is considerable variability among the studies reviewed in terms of the types of genes and techniques used, most of these models revealed a progressive and significant, albeit modest, loss of TH-positive neurons in the SNpc. As can be ascertained from Table 1, the *α*-syn models show progressive cell loss, with protein aggregation, but little evidence for consistent motoric deficits. The use of the *α*-syn A53T rat model did produce significant TH-positive cell loss (at about 60%) and concomitant motor deficits, but this occurred in only 25% of the rats receiving this mutated gene. The LRRK2 model produced both a level of cell loss comparable to early-stage PD and motor deficits in the open-field maze, but there is no evidence of protein aggregation in these animals. In terms of treatments, studies using transgenic PD rats have indicated that parkin can provide a significant level of protection against dopaminergic neuronal loss. Collectively, these studies indicate that the currently used transgenic rat models of PD can provide a useful tool for the early stages of PD, but more work is needed in developing a model that mimics the progressive neuropathology and behavioral deficits that are observed throughout the time span of this disease.

## Figures and Tables

**Table 1:** Summary of studies using genetic rat models of PD. Note that % cell loss SNpc is percentage of cell loss in the substantia nigra, pars compacta, relative to number of cells in the intact, untreated controls; “n.a.” indicates data for percentage loss of SNpc cells was not available; plus sign (+) indicates significant differences and “n.s.” indicates non-significant differences between genetically altered rats and wild-type controls on measures of protein aggregation or behavioral deficits (i.e., stimulant-induced rotational activity, paw placement in cylinder task, and/or spontaneous motor activity in open-field).

Gene	Method	Percentage of cell loss SNpc	Protein aggregation	Behavior deficit	Author year
h*α*-syn, *α*-syn A53T	rAAV	30%–80%	+	+ (in 25% of animals)	Kirik et al. 2002 [12]
h*α*-syn A30P	rAAV	53%	+	n.s.	Klein et al. 2002 [13]
h*α*-syn A30P	HIV-1 Lentivirus	33%	+	None performed	Lo Bianco et al. 2002 [14]
h*α*-syn A53T	HIV-1 Lentivirus	24%	+	None performed	Lo Bianco et al. 2002 [14]
h*α*-syn	rAAV	50%	+	n.s.	Yamada et al. 2004 [15]
h*α*-syn	rAAV2/5	40–60%	+	None performed	Gorbatyuk etal. 2008 [16]
h*α*-syn S129A	rAAV2/5	66–70%	+	None performed	Gorbatyuk etal. 2008 [16]
h*α*-syn S129D	rAAV2/5	n.a.	+	None Performed	Gorbatyuk et al. 2008 [16]
h*α*-syn	rAAV2/6	11–22%	+	None Performed	da Silveira et al. 2009 [17]
h*α*-syn A30P	rAAV2/6	11–22%	+	None Performed	da Silveira et al. 2009 [17]
h*α*-syn WT S129A	rAAV2/6	over 70%	+	None Performed	da Silveira et al. 2009 [17]
h*α*-syn WT S129D	rAAV2/6	n.a.	+	None Performed	da Silveira et al. 2009 [17]
h*α*-syn A30P S129A	rAAV2/6	over 70%	+	None Performed	da Silveira et al. 2009 [17]
h*α*-syn A30P S129D	rAAV2/6	n.s.	+	None Performed	da Silveira et al. 2009 [17]
h*α*-syn A30P	TAT	21.3–31.3%	n.s.	+	Recchia et al. 2008 [18]
h*α*-syn A30P A53T	Microinjection	n.a.	n.s.	+	Lelan et al. 2011 [19]
LRRK2 G2019S	Rad	21.4%	n.s.	None Performed	Dusonchet et al. 2011 [20]
LRRK2 G2019S	TRE-tTA	0%	n.s.	+	Zhou et al. 2011 [21]
